# The molecular biology of disease vectors collection: celebrating the tenth workshop on genetics, molecular biology, and control of insect vectors of tropical diseases (the Entomol)

**DOI:** 10.1186/s13071-026-07557-0

**Published:** 2026-08-03

**Authors:** Sinval Pinto Brandão-Filho, Maria Helena Neves Lobo Silva-Filha, Filipe Dantas-Torres

**Affiliations:** Aggeu Magalhães Institute, Fundação Oswaldo Cruz (Fiocruz), Recife, Brazil

Vector-borne diseases remain at the forefront of emerging public health challenges. A combination of factors has significantly altered the dynamics of these diseases, including ongoing climate change, the expansion of agricultural frontiers, and the globalized transport of people and goods [1]. The growing human incursion into sylvatic environments, for both economic activities and recreation, has expanded the human-zoonotic pathogen interface, increasing the risk of pathogen spillover and spread [2, 3]. Additional challenges include horizontal pathogen transfer among species, the expansion of arboviruses, and the spread of invasive vector species, all of which are facilitated by changing ecosystems [4].

In this context, research on disease vectors has become central and has recently benefited from advances in next-generation sequencing, genomics, and gene-editing tools [5, 6]. These approaches have greatly enhanced our understanding of the molecular mechanisms underlying pathogen-insect interactions, provided innovative methods for studying insect ecology and behavior, and facilitated the development of novel tools for insect control and for assessing emerging vectors and pathogens. Information technologies, coupled with emerging artificial intelligence-based tools, have become essential components of surveillance and vector control strategies [7]. Alongside these advances, a transdisciplinary and integrative view of pathogens, insects, humans, and ecosystems has been necessary to address the complex landscape of vector-borne diseases [8].

The Workshop on Genetics and Molecular Biology of Insect Vectors of Tropical Diseases (known as “Entomol”) has served as a forum for disseminating research on insect vectors and for promoting strategic discussions to address vector-borne disease challenges since its first edition in 2004. In September 2024, its 10th edition was held as a satellite event of the 59th Brazilian Society of Tropical Medicine Congress in São Paulo, marking two decades of biannual editions of this forum. It continues to bring together researchers, students, and surveillance and control professionals working with vectors that transmit diseases such as malaria, leishmaniasis, filariasis, Chagas disease, spotted fever rickettsioses, and arboviruses.

Since its inception in 2004, Entomol participants have addressed historic and emerging vector-related issues, spanning endemic disease challenges in affected countries and emergent global threats. Across ten workshop editions, high-quality work has been presented by Brazilian research teams and invited international participants. The success of the meeting and the significant participation of international speakers led colleagues from the Society for Vector Ecology (SOVE) to establish the Brazilian chapter of SOVE during the 2010 edition (Entomol 4). This was followed by subsequent editions from 2012 to 2018, culminating in the establishment of the Latin American SOVE after the international SOVE congress in Manaus in 2019.

Studies presented and discussions initiated at the Entomol have contributed to knowledge production in this strategic field, specialized personnel training, and the formation of research networks. Key topics discussed have included: conventional and microbial insecticides within integrated vector control; innovative trapping and related methods for surveillance and control based on insect biology and behavior; molecular tools and artificial intelligence applied to taxonomy; platforms for rearing and infecting major vector species whose studies have been delayed by the lack of colonies; cutting-edge advances in biochemistry and molecular biology that enhance knowledge of vector biology and vector-pathogen interactions; genomic surveillance and dynamics of recently introduced arboviruses, such as chikungunya and Zika, in vectors; characterization and functional studies of vector microbiota; strategies for genetically modified mosquitoes; and large-scale deployment of *Aedes aegypti* infected with *Wolbachia* to reduce arbovirus transmission. In addition, several persistent challenges, such as yellow fever [3] and spotted fever [9] outbreaks, the expansion of dengue, chikungunya [10], Oropouche fever [11], and leishmaniasis [12], and changes in the eco-epidemiological profile of Chagas disease [13], have been frequently discussed during Entomol meetings because they may threaten the control achievements obtained in the past.

The collection Molecular Biology of Disease Vectors includes papers presented at the 10th Entomol**,** along with other contributions on the molecular biology, surveillance, and control of disease vectors. It brings together numerous articles covering molecular biology (e.g., population genetics, phylogenetics and evolution, molecular taxonomy, and vector-pathogen interactions), as well as surveillance and control of disease vectors, such as mosquitoes, phlebotomine sand flies, ticks, triatomine bugs, and biting midges (https://link.springer.com/collections/gffeegbeeg). We hope that the Entomol will continue to strengthen the integration of research groups working on arthropod vectors and associated diseases, foster the exchange of new knowledge and technological innovations in this important field of science, and support public policies for control and surveillance in Brazil and other countries worldwide.

## Data Availability

Not applicable.
